# A scalable approach to investigating sequence-to-function predictions from personal genomes

**DOI:** 10.1038/s41592-026-03124-8

**Published:** 2026-06-08

**Authors:** Anna E. Spiro, Xinming Tu, Yilun Sheng, Alexander Sasse, Rezwan Hosseini, Maria Chikina, Sara Mostafavi

**Affiliations:** 1 Paul G. Allen School of Computer Science and Engineering, University of Washington, Seattle, WA USA; 2 Department of Computational and Systems Biology, University of Pittsburgh, Pittsburgh, PA USA; 3 Present Address: Genentech, South San Francisco, CA USA

**Keywords:** Computational models, Machine learning, Genomics, Software

## Abstract

Sequence-to-function (S2F) models can evaluate arbitrary DNA sequences, yet they struggle to fully capture inter-individual variation in gene expression. We introduce SAGE-net, a scalable framework for training and evaluating S2F models using personal genomes. While personal genome training improves gene expression prediction accuracy for held-out individuals, performance gains arise primarily from identifying predictive variants rather than learning a *cis*-regulatory grammar that generalizes across loci. Scalable software will be critical to advancing S2F models for personal genomics.

## Abstract (web summary)

Modeling the effect of DNA sequence variation on phenotypes such as gene expression faces unique challenges when deciphering inter-individual variation. This study presents a scalable and efficient sequence-to-function modeling framework for personal genome analysis.

## Main

Sequence-to-function (S2F) deep neural networks have recently emerged as powerful tools for predicting how genomic DNA maps to cell-type-specific regulatory functions^1–7^. Unlike statistical genetics models that rely on large cohorts to associate observed genetic variation with gene expression, S2F models take a mechanistic approach to learning gene regulation. This allows them to predict the impact of unseen genetic variants while also providing insights into the regulatory grammar encoded in genomic DNA that drives their predictions. By bridging prediction and explanation, S2F models offer a coherent framework for interpreting genetic variants in personal genomes.

However, recent studies, including our own, have revealed that current S2F models trained on a single reference genome (which we refer to as reference-S2F) struggle to accurately predict gene expression from personal genomes^8,9^. This observation suggests that while reference-S2F models capture certain aspects of *cis*-regulatory grammar that drive gene expression differences across genes, their ability to grasp the finer rules governing inter-individual variation in gene expression remains limited. Closing this gap requires higher-resolution training data, and a compelling approach is to train or fine-tune S2F models on cohort datasets with paired whole-genome sequencing (WGS) and RNA-seq, allowing them to learn how subtle genetic variation impacts gene expression^10–13^. Initial efforts have begun to explore this direction, but a major bottleneck remains in computational scalability, which impedes rapid cycles of experimentation and result analysis.

Here we developed a scalable framework for training S2F models on personal genomes, which we call SAGE-net (‘small and good enough’). Our approach addresses important gaps in existing software by incorporating key components: (1) an ‘on-the-fly’ personal dataset that converts personal genomes into one-hot-encoded matrices for S2F training; (2) a contrastive learning architecture that emphasizes inter-individual variation; and (3) compact convolutional neural networks (CNNs) that deliver performance comparable to fine-tuning large S2F models at substantially lower computational cost.

**Extended Data Fig. 1:**
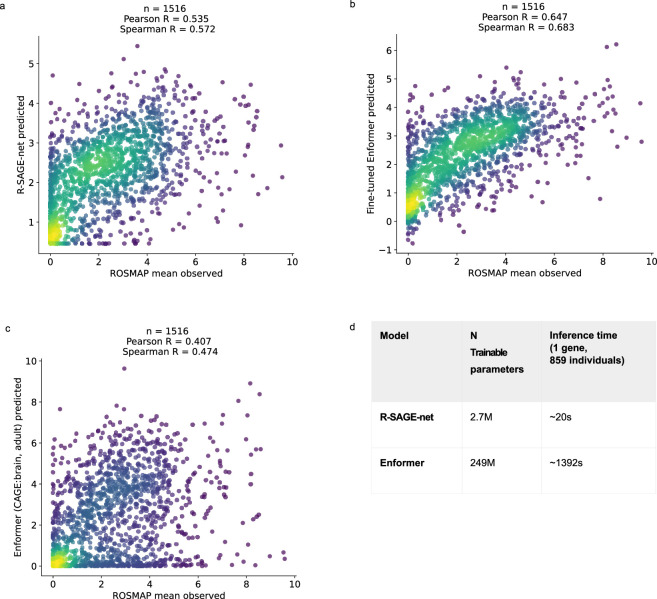
Comparisons between r-SAGE-net and Enformer. (**a**) R-SAGE-net trained on genes in Enformer’s training set (*n* = 15,335) and evaluated on genes in Enformer’s test set (*n* = 1516). R-SAGE-net is trained and evaluated on 40 kb reference sequence and average log-transformed gene expression across ROSMAP individuals. Scatterplot is colored by density. (**b**) Fine-tuned Enformer evaluated on Enformer’s test set genes. See Methods for fine-tuning procedure. Evaluation is done with the same length input as in Enformer training (196,608 bp). (**c**) Enformer evaluated on Enformer’s test set genes without fine-tuning. Insead of fine-tuning, we select the representative track (‘CAGE:brain, adult’). (**d**) Comparison between r-SAGE-net and Enformer model sizes and inference times. Both models are evaluated on one NVIDIA RTX A4000 GPU.

First, we compared the performance of this reference-only-trained CNN (r-SAGE-net) with that of Enformer in predicting mean (averaged across individuals) cortex gene expression from unseen genomic locations (Extended Data Fig. 1a,b), using RNA sequencing (RNA-seq) data from the ROSMAP (Religious Orders Study and Rush Memory and Aging Project) cohort^14^. While Enformer outperforms r-SAGE-net, r-SAGE-net achieves strong performance while reducing inference time by 70-fold (Extended Data Fig. 1d).

**Fig. 1:**
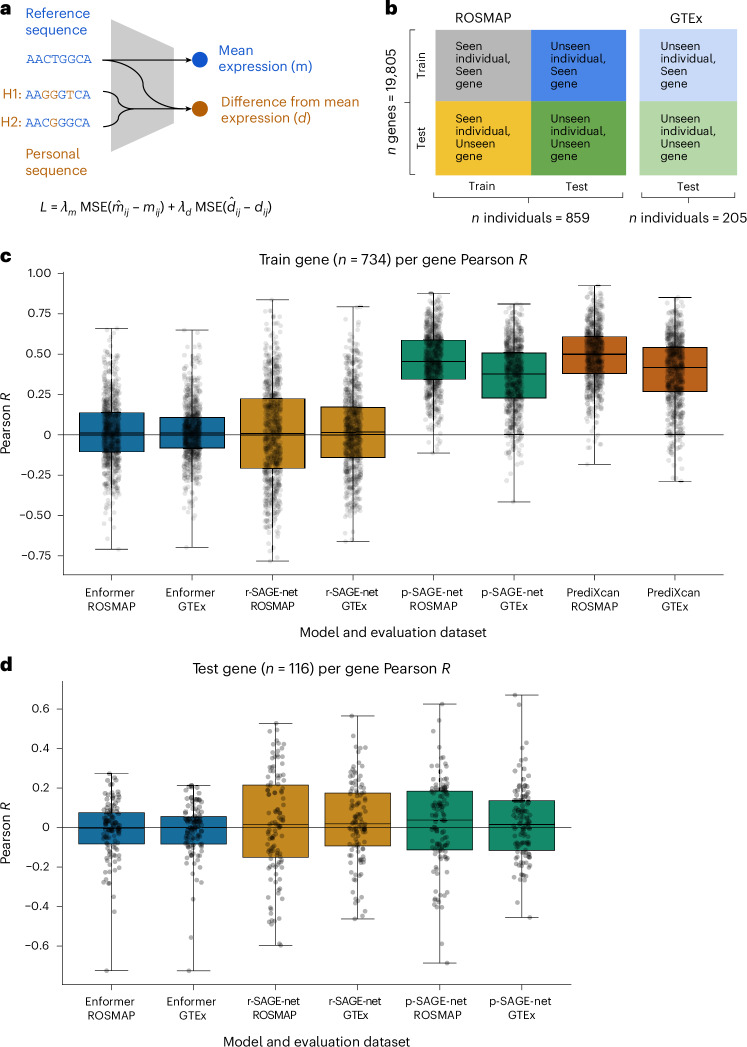
P-SAGE-net model and its performance on personal gene expression prediction. **a**, Schematic of p-SAGE-net. For a given data point (one gene from one individual), the model takes as input three 40-kb one-hot-encoded sequences of genomic DNA centered at gene transcription start site: reference sequence and personal sequences from the individual’s two haplotypes (in practice, we use phased WGS data). The model uses reference sequence to predict mean gene expression (measured as the average log-transformed gene expression across individuals in a cohort, here ROSMAP), and a combination of reference and personal sequence to predict personal difference from mean gene expression. The loss function (*L*) combines the MSE loss of ‘mean’ and the mean squared error (MSE) loss of ‘difference’ using a hyperparameter (*λ*). **b**, Data splits across genes and individuals for the ROSMAP and GTEx datasets. For clarity, the validation split is not shown in the figure. **c**, P-SAGE-net compared to baselines on per-gene Pearson correlation (measured across 85 (ROSMAP) and 205 (GTEx) unseen individuals) for 734 seen genes from the top 1,000 gene set. For boxplots here and in **d**, each dot is one gene, boxplots are centered at median with top and bottom showing interquartile range and whiskers extending to minimum and maximum. **d**, Same comparisons as in **c**, but for 116 unseen genes from the same top 1,000 gene set. Note that as PrediXcan is trained per-gene, it cannot be used to make predictions for unseen genes and is thus not shown.

Encouraged by this result, we created personal-SAGE-net (p-SAGE-net), which uses a CNN in a contrastive learning framework to decouple the ‘mean’ and the ‘personal’ components of gene expression per locus (Fig. 1a). Following previous work^10,11^, we trained and tested our model on personal genomes. We use cortex RNA-seq from the ROSMAP cohort (*n* = 859 individuals) for training and testing and use the GTEx cohort^15^ (*n* = 205 individuals) as an additional test set (Fig. 1b). For our initial analysis, we use the top 1,000 genes ranked by PrediXcan linear estimate of heritability^16^ (Methods). P-SAGE-net achieved performance comparable to PrediXcan when evaluated on unseen alleles for the training genes (ROSMAP *n* = 85, GTEx *n* = 205 individuals per 734 training genes; Fig. 1c)–a modest but critical benchmark for evaluating model effectiveness. We note that p-SAGE-net achieves similar performance to models that fine-tune Borzoi or Enformer^10,11^.

**Extended Data Fig. 2:**
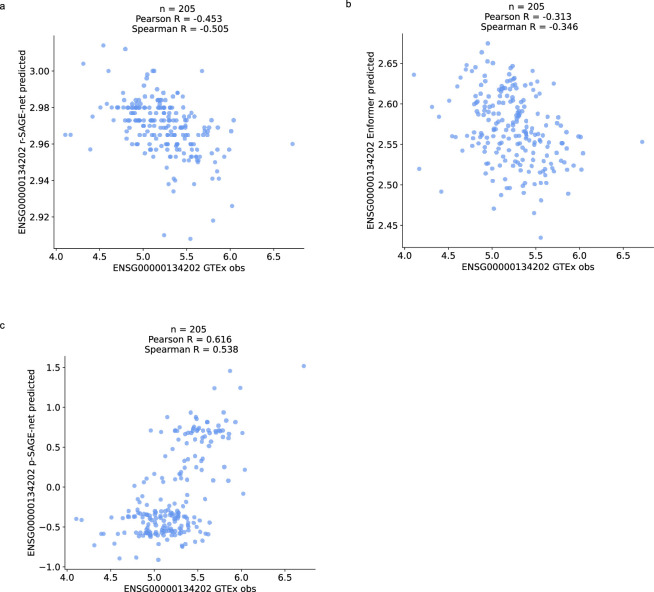
Model performance for GSTM3. (**a**) R-SAGE-net GTEx predicted vs. observed. (**b**) Fine-tuned Enformer GTEx predicted vs. observed. (**c**) P-SAGE-net GTEx predicted vs. observed. Note that the p-SAGE-net model used here was trained on the top 1000 gene set, which includes GSTM3 (but the GTEx individuals shown here were not seen in model training).

**Extended Data Fig. 3:**
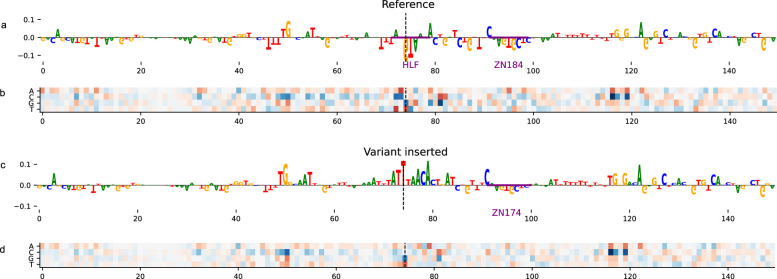
P-SAGE-net ISM for GSTM3. (**a**) Zero-centered ISM for a 150 bp window around the variant of interest (chromosome 1, hg38 position 109731286 G → T), shown for the reference sequence. Motifs shown are from seqlets matched to the HOCOMOCO v12 database by TOMTOM with p < 0.05. (**b**) Same ISM as in (**a**), but shown for all bases instead of only reference sequence. White represents value=0, red shows positive attributions, blue shows negative attributions. (**c**) Same ISM as in (**a**), but done for reference sequence with variant inserted (T instead of G at 109731286). (**d**) Same ISM as in (**c**), but shown for all bases instead of only reference sequence.

**Extended Data Fig. 4:**
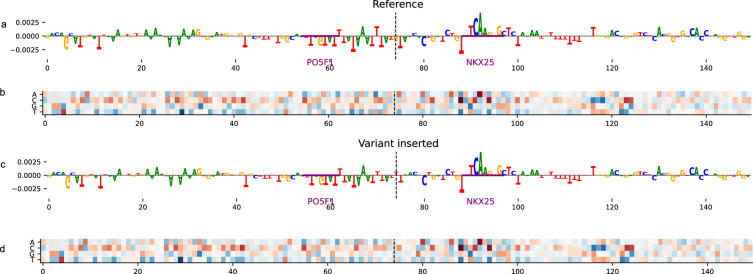
R-SAGE-net ISM for GSTM3. Same analysis as Extended Data Fig. 3, but using r-SAGE-net instead of p-SAGE-net. (**a**) Zero-centered ISM for a 150 bp window around the variant of interest (chromosome 1, hg38 position 109731286 G → T), shown for the reference sequence. Motifs shown are from seqlets matched to the HOCOMOCO v12 database by TOMTOM with p < 0.05. (**b**) Same ISM as in (**a**), but shown for all bases instead of only reference sequence. White represents value=0, red shows positive attributions, blue shows negative attributions. (**c**) Same ISM as in (**a**), but done for reference sequence with variant inserted (T instead of G at 109731286). (**d**) Same ISM as in (**c**), but shown for all bases instead of only reference sequence.

**Extended Data Fig. 5:**
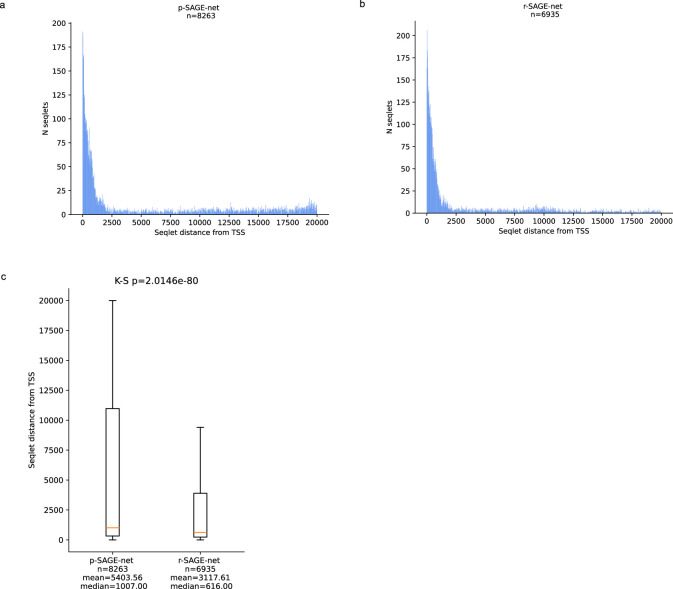
Seqlet distances from TSS for p-SAGE-net vs. r-SAGE-net. (**a**) Distribution of p-SAGE-net seqlet distances from TSS. Analysis shown (combined) for high performance genes in model training set (n=466 genes with GTEx per-gene Pearson correlation > 0.3). We approximate ISM values using zero-centered gradients and then identify seqlets with p < 0.005 (see Methods). (**b**) Same analysis as (**a**), but with r-SAGE-net instead of p-SAGE-net. (**c**) Comparison between seqlet distances from gene TSS for p-SAGE-net and r-SAGE-net. P-value is from the two-sided two-sample Kolmogorov-Smirnov test for goodness of fit. Boxplots are centered at median with top and bottom showing interquartile range and whiskers extending to minimum and maximum.

We then examined whether p-SAGE-net learns additional *cis*-regulatory grammar to more accurately predict gene expression variation across individuals compared to reference-S2F models. Using in silico mutagenesis (ISM), we identified cases where p-SAGE-net captures regulatory grammar underlying inter-individual expression differences–patterns often missed by reference-S2F models. For instance, for the *GSTM3* gene, reference-S2F models incorrectly predict expression changes with a negative correlation to the observed data, whereas the personal counterpart model produces accurate, positively correlated predictions (Extended Data Fig. 2). ISM analysis for p-SAGE-net near the Susie-fine-mapped causal variant^17^ reveals a repressive motif, matching the transcription factor HLF, which is disrupted by the variant (Extended Data Fig. 3)–a pattern absent in both r-SAGE-net (Extended Data Fig. 4) and Enformer (Supplementary Fig. 1). Beyond analyzing specific examples, we also systematically compared seqlets–short sequence motifs driving model predictions–between p-SAGE-net and its reference-only counterpart (Extended Data Fig. 5 and Methods), and found that training on personal genomes helps mitigate models’ previously observed under-reliance on distal variants^18^.

**Extended Data Fig. 6:**
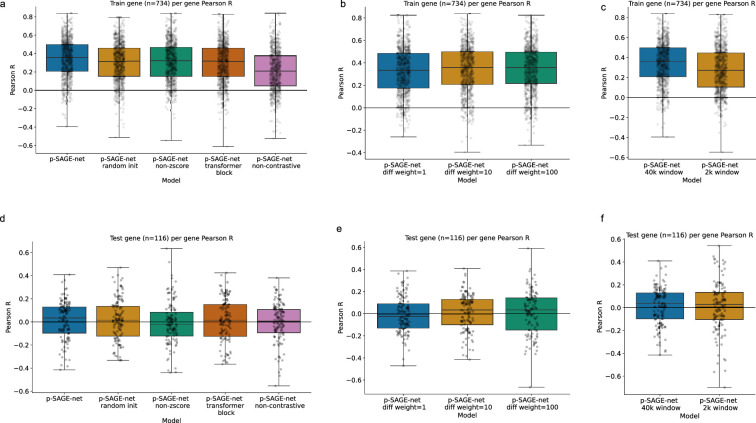
Model ablation analyses. (**a**) Comparison between best model (p-SAGE-net) and variations: randomly initializing model weights instead of initializing from r-SAGE-net (‘random init’), using personal expression difference instead of z-score for model difference output (‘non-z-score’), replacing the last convolutional block with a transformer block (‘transformer block’), and predicting personal gene expression from personal sequence, without the contrastive approach (‘non-contrastive’). See Methods for details on each variation. Each model is trained on 689 ROSMAP training individuals for 734 training genes from the top 1000 gene set and evaluated on 205 GTEx individuals for the same gene set. For boxplots here and in (**b**), (**c**), (**d**), (**e**), and (**f**), each dot is one gene, boxplots are centered at median with top and bottom showing interquartile range and whiskers extending to minimum and maximum. (**b**) Modifications to loss function hyperparameters. For each weight on the ‘difference’ portion of the loss function (diff weight = 1, 10, 100), the weight on the ‘mean expression’ portion of the loss function = 1–so when diff weight = 1, the two portions of the loss function are equally weighted. The difference weight selected for all other analyses is 10. Model training and evaluation gene and individual sets are the same as in (**a**). (**c**) Comparison between models with 40 kb vs. 20 kb input window. Window size applies to both model training and evaluation. Model training and evaluation gene and individual sets are the same as in (**a**). (**d**) Same analysis as (**a**), but shown for unseen test genes (n=116). (**e**) Same analysis as (**b**), but shown for unseen test genes. (**f**) Same analysis as (**c**), but shown for unseen test genes.

We next evaluated p-SAGE-net’s ability to predict unseen alleles from unseen genes. This represents an important but notably more challenging evaluation, as it requires the model’s learned *cis*-regulatory grammar to generalize across both loci and individuals. Success here would demonstrate true sequence comprehension and support the broader goal of extending prediction to de novo variants and previously uncharacterized loci; however, as also previously shown for other personal-S2F models^10,11^, we observed that p-SAGE-net does not generalize to alleles in unseen genes (Fig. 1d). We explored variations to model initialization, loss function, input sequence length and architecture, and while some of these modifications affected seen gene per-gene performance, none allowed the model to generalize to unseen alleles in unseen genes (Extended Data Fig. 6.)

**Fig. 2:**
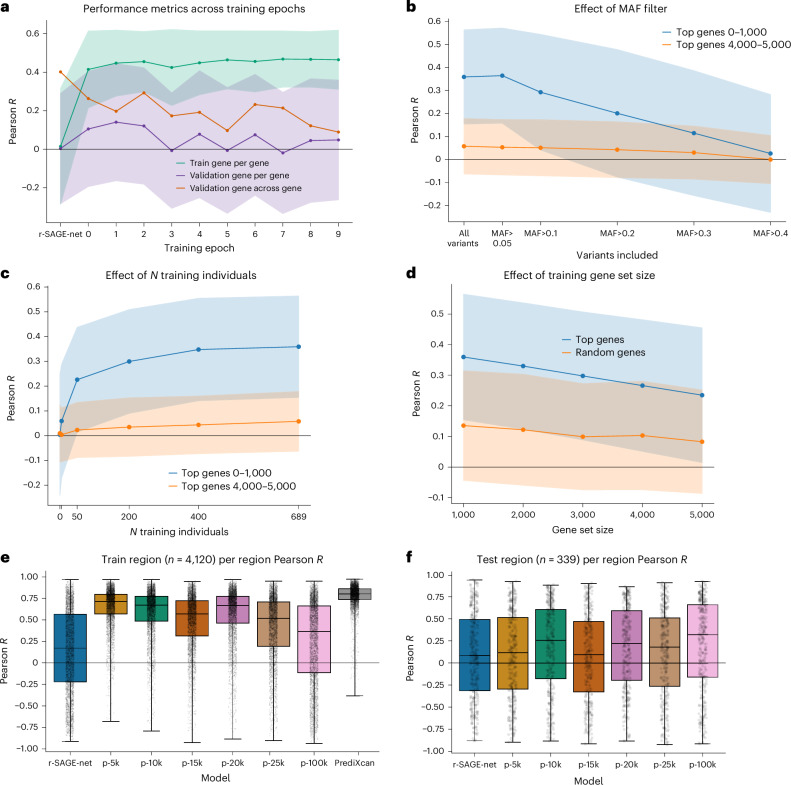
P-SAGE-net performance across experimental conditions and molecular modalities. **a**, Three different metrics (train gene per-gene Pearson *R*, validation gene per-gene Pearson *R*, validation gene across-gene Pearson *R*) across ten training epochs for p-SAGE-net. As p-SAGE-net is initialized from r-SAGE-net, r-SAGE-net metrics are shown before epoch 0. Training genes are the same 734 as in Fig. 1c, validation genes are 114 genes from the same top 1,000 set. For train gene per-gene and validation gene per-gene, correlations are across 85 validation individuals. Plots here and in **b**–**d** show median Pearson *R* values with shading showing ±s.d. **b**, P-SAGE-net evaluated with MAF filters for two different gene sets. MAF is calculated using training individuals and is used in model evaluation, not model training—the model is trained using all variants. At each MAF threshold, the model is evaluated on all 205 (unseen) GTEx individuals for each gene present in the model training set: 734 genes for the 0–1,000 gene set, 756 genes for the 4,000–5,000 gene set. **c**, P-SAGE-net trained on different numbers of training individuals for two different gene sets (the same sets as in **b**). Models are trained on different numbers of ROSMAP training individuals, 0, 5, 50, 200, 400 and 689 (all), and evaluated on 205 GTEx individuals. **d**, P-SAGE-net trained on different numbers of genes, with genes added either by PrediXcan rank or randomly. For ‘top genes’, gene set size *n* = 1,000 means that the model has been trained on all genes in the top 1,000 set that are in the training chromosomes split (*n* = 734). The gene set size is then increased up to 5,000 by adding genes in descending rank, up to *n* = 3,675 training genes for the top 5,000 gene set. For ‘random genes’, the 1,000 gene set is randomly selected from the top 5,000 genes and then increased by randomly adding genes within this top 5,000 gene set, such that larger sets contain all genes included in smaller sets. For both ‘top genes’ and ‘random genes’, models are evaluated on 205 GTEx individuals for training genes in the relevant 1,000 gene set (*n* = 734 genes for top genes, *n* = 756 genes for random genes). **e**, DNAm R-SAGE-net, p-SAGE-net (with varying training region set sizes) and PrediXcan performance across 54 test individuals for 4,120 train regions from the top 5,000 region set. R-SAGE-net was not trained on personal genomes. P-SAGE-net models were trained on 513 training individuals for training regions from each region set (each of which include these 4,120 regions) for a single epoch. PrediXcan is trained per-region on the same 513 training individuals. For boxplots here and in **f**, each dot is one region, boxplots are centered at median with top and bottom showing interquartile range and whiskers extending to minimum and maximum. **f**, Same models as in **e**, but for 339 unseen test regions. Note that as PrediXcan is trained per-region, it cannot be used to make predictions for unseen regions and is thus not shown.

We also noticed that p-SAGE-net’s performance on predicting differences between genes is worse than r-SAGE-net’s (Supplementary Fig. 2), suggesting that the model ’forgets’ generalizable cross-gene *cis*-regulatory grammar during personal genome training. Indeed, with each epoch, unseen gene mean expression prediction performance progressively declined (Fig. 2a), likely due to the model overfitting to the training genes.

Our flexible on-the-fly personal dataset, which constructs sequences directly from variant call format files given a specified minor allele frequency (MAF) threshold, readily enables examining model performance on variants filtered by MAF. We assessed two gene sets: the top 1,000 genes and the top 4,000–5,000 genes (as ranked by PrediXcan). We included this lower-ranked gene set out of concern that when heritability is estimated using population-level statistical models, genes appearing to have higher heritability may actually be those primarily driven by a single common causal variant with a large effect. By applying MAF filters to exclude increasingly common variants, we see that low-frequency variants (MAF < 0.05) contribute little to model predictions for either gene set, and more broadly, that gene sets with different ranges of statistical heritability show different patterns in model performance (Fig. 2b). We also test the effect of only including single-nucleotide variants (SNVs) in model evaluation (instead of including all variants) (Supplementary Fig. 3) and find a small performance decrease for the more heritable gene set only (two-sided Wilcoxon signed-rank test, *P* = 0.0228).

We additionally examined training sample size requirements using the same two gene sets. While model performance for the top 1,000 gene set plateaued after approximately 400 training individuals, the 4,000–5,000 gene set exhibited a more gradual performance increase, suggesting that these genes require larger datasets to capture meaningful regulatory patterns (Fig. 2c). This aligns with our intuition that ‘easier’ genes (those whose expression can be well-predicted from just a few variants) require fewer training samples, whereas ‘harder’ genes demand more data to uncover the larger set of weaker factors driving inter-individual variation.

We hypothesized that fine-tuning p-SAGE-net on many genes (>1,000) should provide additional training data for learning generalizable regulatory grammar, leading to improved performance as the number of training genes increases. However, when we add more training genes (either randomly (‘random genes’) or ranked by decreasing PrediXcan performance (‘top genes’)) we observe a drop in performance for the core smallest set of genes (Fig. 2d), even with increased model capacity (Supplementary Fig. 4), suggesting that the model is not learning a generalizable regulatory grammar.

Gene expression is a complex process involving the interplay of epigenomic, transcriptional and post-transcriptional mechanisms. To investigate the effects of personal genome training in a simpler setting, we apply our p-SAGE-net framework to DNA methylation (DNAm) data. DNAm is a key epigenomic mark and is currently available at cohort scale using array-based platforms (for example, 450K Illumina array data available in ROSMAP, *n* = 634). Understanding how genetic sequence encodes DNAm variation remains an important open problem, especially given DNAm’s role as a mediator of GWAS signals^19,20^.

**Extended Data Fig. 7:**
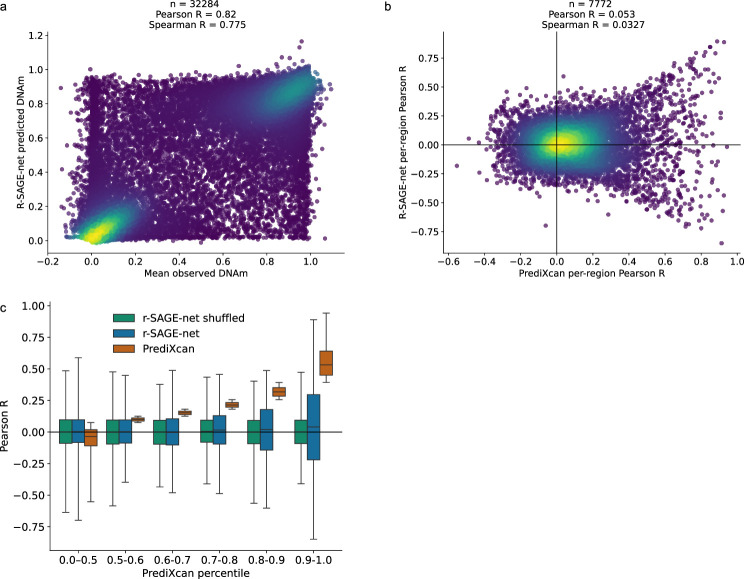
DNAm r-SAGE-net performance across regions and per-region. (**a**) R-SAGE-net predicted DNAm from reference sequence vs. population mean observed DNAm for 32,284 test regions. Here and in (**b**), scatterplot is colored by density (**b**) R-SAGE-net vs. PrediXcan performance per-region across 54 test individuals for 7772 test regions from 100,000 randomly sampled regions. (**c**) Same evaluation as (**b**) (n=7222 test regions), shown binned by PrediXcan Pearson R percentile. ‘R-SAGE-net shuffled’ contains per-region Pearson correlation between r-SAGE-net predicted DNAm and observed DNAm with shuffled columns (shuffled individual labels). Boxplots are centered at median with top and bottom showing interquartile range and whiskers extending to minimum and maximum.

We first evaluated the performance of a reference-trained model in predicting DNAm across genomic loci, and observed good model performance (Pearson *R* = 0.82; Extended Data Fig. 7a); however, when assessing its ability to predict inter-individual variation, we observed a pattern similar to what we see in gene expression: reference-trained models struggle to correctly predict the direction of genetic effects (Extended Data Fig. 7b), although unlike in the case of gene expression, reference model performance does slightly increase with increased linear heritability (Spearman correlation, *ρ* = 0.0327, *P* = 3.94 × 10^−3^; Extended Data Fig. 7b,c).

**Extended Data Fig. 8:**
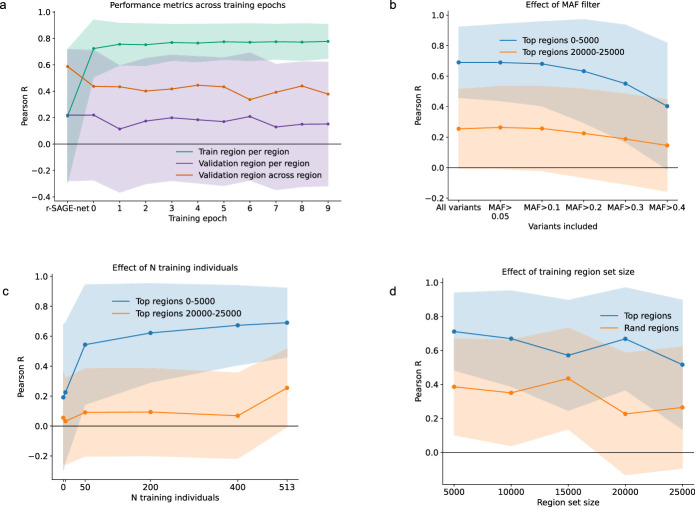
DNAm p-SAGE-net performance across training epoch, MAF filter, number of training individuals, and region set size for seen regions. (**a**) Same analysis as Fig. 2a, but for DNAm instead of gene expression. Results are show for the (PrediXcan-ranked) top 5000 region set. This region set contains 4120 train regions and 541 validation regions. Results are shown for 67 unseen validation individuals. Plots here and in (**b**), (**c**), and (**d**) show median Pearson R values with shading showing +/- standard deviation. (**b**) Same analysis as Fig. 2b, but for DNAm instead of gene expression. Results are shown for different evaluation MAF thresholds for seen training regions from the top 0-5000 region set (4120 regions) and seen training regions from the top 20,000-25,000 region set (4104 regions). Results are shown for 54 unseen test individuals. (**c**) Same analysis as Fig. 2c, but for DNAm instead of gene expression. Region sets and test individuals are the same as in (**b**). 513 is the total number of training individuals for this data. (**d**) Same analysis as Fig. 2d, but for DNAm instead of gene expression. The evaluation shown here is for training regions from the top 5000 region set: 4120 regions for ‘Top regions’, 4104 regions for ‘Random regions’.

We then leveraged the scalability of our training suite to train p-SAGE-net on personal DNAm. As in the case of expression, personal genome training greatly improves per-region correlation for seen regions (Fig. 2e), but this seen-region performance decreases with increasing training set size. We see similar trends across training and evaluation settings for DNAm as we do for gene expression (Extended Data Fig. 8).

**Extended Data Fig. 9:**
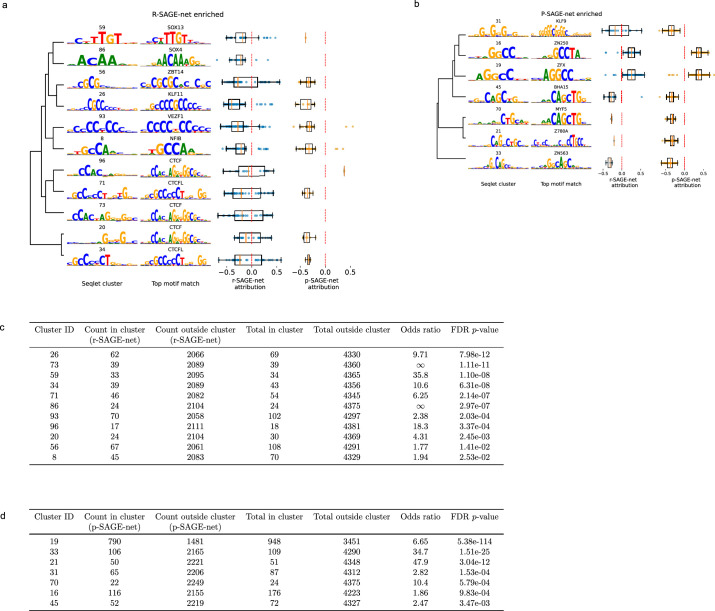
DNAm attribution enrichment analysis. (**a**) Global attribution analysis showing seqlet clusters enriched for r-SAGE-net. From left to right, columns show: seqlet clusters, cluster matches to motif database (motifs plotted are position probability matrices scaled by information content), distributions of seqlet mean attributions per cluster for r-SAGE-net, and distributions of seqlet mean attributions per cluster for p-SAGE-net. The clusters shown are those that are enriched significantly for r-SAGE-net (one-sided Fisher’s exact test, FDR-adjusted p < 0.05) and match significantly to a known database motif (TOMTOM q < 0.05); see Methods for details. For boxplots here and in (**b**), each dot is one seqlet, boxplots are centered at median with left and right showing interquartile range and whiskers extending to minimum and maximum. (**b**) Same analysis as (**a**), but for seqlet clusters enriched for p-SAGE-net (training region set size=100k). (**c**) Details of clusters shown in (**a**). (**d**) Details of clusters shown in (b).

**Extended Data Fig. 10:**
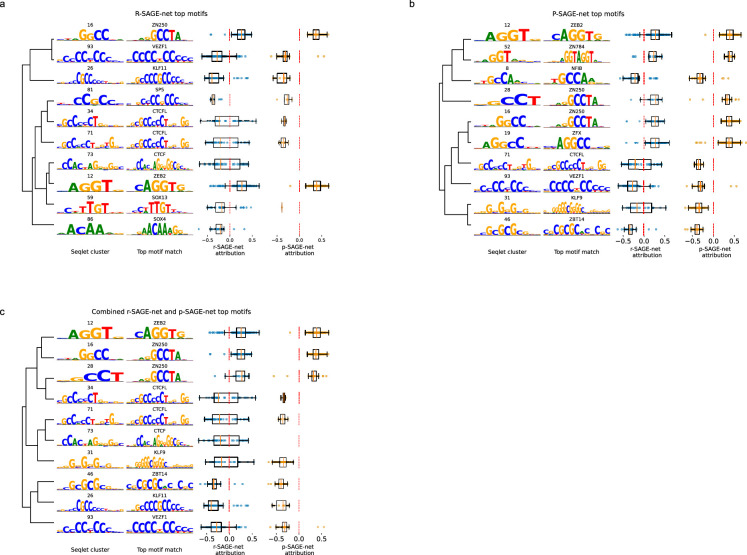
Additional views of DNAm attribution analyses. (**c**) Global attribution analysis showing top seqlet clusters for r-SAGE-net. These 10 clusters are selected based on number of seqlets, mean absolute seqlet attribution, and significance of match (see Methods for details). Figure layout is the same as Extended Data Fig. 9. For boxplots here and in (**b**) and (**c**), each dot is one seqlet, boxplots are centered at median with left and right showing interquartile range and whiskers extending to minimum and maximum. (**b**) Same analysis as (**a**), but shown for p-SAGE-net top clusters. (**c**) Same analysis as (**a**), but shown combined for r-SAGE-net and p-SAGE-net.

We next evaluated p-SAGE-net on the more challenging task of predicting DNAm variation across unseen individuals in unseen regions. Unlike with gene expression, personal genome training on DNAm (especially with increasing training region set size) enabled improved generalization to unseen genomic regions (*P* = 4.96 × 10^−4^, two-sided Wilcoxon signed-rank test between r-SAGE-net and p-SAGE-net trained on 100,000 regions; Fig. 2f). To interpret what the model has learned, we performed a global attribution analysis by clustering seqlets and matching them to a known motif database (Extended Data Figs. 9 and 10 and Methods). These results suggest that, given sufficient sequence diversity, models can begin to capture subtle patterns of inter-individual DNAm variation.

In summary, our findings reveal critical limitations (namely, model inability to generalize to unseen genes) that are broadly relevant to personal-sequence-to-expression modeling efforts, as well as promising results in the context of epigenomic data. We envision pre-training on random DNA sequences^21^ and integrating epigenomic data modalities with WGS and RNA-seq as promising directions toward a more comprehensive understanding of inter-individual expression differences.

While deep-learning models have yet to surpass linear methods such as PrediXcan in gene expression prediction accuracy, we view this as a transitional phase (not proof against using deep models for these tasks, but rather a sign that tools, architectures and training paradigms are still evolving). The strength of deep models lies in their ability to evaluate arbitrary DNA sequences, including rare and de novo variants, in diverse genomic contexts. This capability is critical for moving beyond well-characterized cohorts and tissues toward a more mechanistic, sequence-based understanding of gene regulation.

## Methods

### WGS and RNA-seq datasets ROSMAP

We use *n* = 859 individuals with available WGS and RNA-seq (dorsolateral prefrontal cortex) from the ROS and MAP cohort studies^14^, as in our previous work^9^. We take log(TPM+1) and preprocess by regressing out known covariates and top expression principal components (PCs), following the process recommended by recent work^22^. We regress out top ten expression PCs as well as the following set of technical and phenotype covariates (as carried out in previous work)^9^: batch, estimated library size, pass filterreads aligned, percent coding bases, percent intergenic bases, percent pass filter reads aligned, percent ribosomal bases, percent untranslated region bases, percent duplication, median 3′ bias, median 5′ to 3′ bias, median coefficient of variation coverage, RNA integrity numberscore, postmortem interval, age of death, sex and top three genotype PCs (from PLINK^23^). We retain gene means. As in previous work^9^, we use phased WGS data. The variant call files for WGS data from the ROSMAP in variant call format were obtained from the Synapse repository (syn117074200). The coordinates of variant calls (GRCh37) were converted to GRCh38 coordinates using the Picard LiftoverVcf tool (http://broadinstitute.github.io/picard). Eagle software2 v.2.4.1 was used to phase the genotypes with the default setting.

### WGS and RNA-seq datasets GTEx

We use *n* = 205 individuals with available WGS and RNA-seq (cortex), GTEx V8. As with ROSMAP, we take log(TPM+1) (from GTEx_Analysis_2017-06-05_v8_RNASeQCv1.1.9_gene_tpm.gct) and regress out the top ten expression PCs, known technical and phenotype covariates (from GTEx_Analysis_v8_Annotations_SampleAttributesDS.txt) and top three genotype PCs. We use phased WGS data from GTEx_Analysis_2017-06-05_v8_WholeGenomeSeq_838Indiv_Analysis_Freeze.SHAPEIT2_phased.vcf.gz.

### DNA methylation dataset ROSMAP

We use *n* = 634 individuals with available WGS and DNAm (dorsolateral prefrontal cortex, 450K Illumina array data) from the ROSMAP cohort. Preprocessing steps are described elsewhere^24^. In brief, after modality-specific DNAm normalization steps, the effects of ancestry, cell-type composition and ‘hidden factors’ (PCs) are regressed out from the DNAm.

### Gene sets

From all genes in the ROSMAP expression data, we filter using annotations from Gencode Release 27 (GRCh38.p10). We filter by gene_type = ‘protein_coding’, feature = ‘gene’, and seqname in chr 1-22 or X, Y, yielding 19,805 unique Ensemble gene IDs. We use TSS definitions from Gencode Release 27. We use the chromosome split: train chromosomes = range (1, 17), validation chromosomes = [17, 18, 21, 22], test chromosome s= [19, 20], which allocates 14,786 genes (78%) to train, 2,126 (11%) to validation and 2008 (11%) to test. These same chromosome splits are also used in the DNAm analysis. We include performance results split by chromosome in Supplementary Fig. 5.

### Enformer gene sets

We downloaded Enformer’s human gene splits from: https://console.cloud.google.com/storage/browser/basenji_barnyard/data. We consider a gene to be in Enformer’s ‘train’, ‘validation’, or ‘test’ set only if our entire input window (TSS − 20,000 to TSS + 20,000) falls within an Enformer ‘train’, ‘validation’, or ‘test’ window. This yields 15,335 train genes, 1,252 validation genes and 1,516 test genes.

### Individual sets

For the gene expression analysis, ROSMAP individuals are split randomly into train (*n* = 689), validation (*n* = 85) and test (*n* = 85) sets to achieve an 80%/10%/10% split. GTEx individuals (*n* = 205) are only used in model evaluation, not in training. For the DNAm analysis, ROSMAP individuals are split randomly into train (*n* = 513), validation (*n* = 67) and test (*n* = 54) sets.

### PrediXcan

We train one PrediXcan model (implemented using scikit-learn ElasticNet) for each of the 19,805 genes on 689 ROSMAP training individuals. We set the input window to be 40 kb, centered on gene TSS. We set l1_ratio (which determines the balance between L1 and L2 penalties within the elastic net penalty) to 0.5 and use ROSMAP validation individuals (*n* = 85) to select α (the weight on the elastic net penalty) for each gene using ElasticNetCV. We use Pearson *R* for ROSMAP validation individuals to create the ranked gene list used throughout our analyses. We use a MAF threshold of 0.01. We use the same procedure to train PrediXcan models for the DNAm analysis, except we use an input window of 10 kb. A 10-kb input window is chosen because it yields similar performance to a 40-kb input window with decreased computational cost (Supplementary Fig. 6), thus allowing us to investigate more regions.

### Sequence inputs

To construct sequence inputs to the S2F models, we use the package pysam to insert all variants (or, those with MAF above a specified threshold) into reference sequence centered on gene TSS (or probe position, for the DNAm analysis). Our personal dataset defaults to inserting all variants (SNVs and indels), but this can be specified by the user (and we vary this for Supplementary Fig. 3). For our gene expression analysis, for r-SAGE-net and p-SAGE-net we use a 40-kb input window, and for Enformer we use Enformer’s full 196,608-bp input window. If a gene is on the negative strand, we take the reverse-complement after one-hot encoding. Including this reverse-complementing in model training and evaluation improved model performance on predicting mean expression but not on predicting differences across individuals (Supplementary Fig. 7). For the DNAm analysis, the procedure for creating sequence inputs is the same, except we use a 10-kb input window (see Supplementary Fig. 6. for window size comparisons).

### R-SAGE-net training

We use Pytorch for all S2F model training. R-SAGE-net is trained on reference sequence and mean expression (averaged across ROSMAP training individuals) for *n* = 14,786 training genes (expression) or *n* = 346,138 training regions (DNAm).

For the hyperparameter tuning process, we grid search over:

- first_layer_kernel_number = (256, **900**): input channels for the first convolutional layer
- int_layers_kernel_number = (**256**, 512, 900): input and output channels for all convolutional layers after the first
- first_layer_kernel_size = (25, **10**): kernel size for the first convolutional layer
- int_layers_kernel_size = (**5**): kernel size for all convolutional layers after the first
- n_conv_blocks = (**5**, 8): number of convolutional blocks (convolutional layer, activation) with dilatio*n* = 1
- pooling_size = (25, **10**, 5): pooling kernel size
- pooling_type = (‘max’, **‘avg’**): pooling kernel type
- n_dilated_conv_blocks = (**0**, 3, 5): number of convolutional blocks with dilation ≠ 1
- dropout = (**0**, 0.2): dropout in fully connected layers
- h_layers = (2, **1**): number of hidden layers
- increasing_dilation = (**False**, True): whether to exponentially increase dilation in dilated_conv_layers (or keep at 2)
- batch_norm = (**True**, False): whether to add batch normalization at the beginning of each convolutional block
- hidden_size = (**256**, 512, 900): number of nodes in fully connected layers
- learning_rate = (**5** × **10**^**−4**^, 1 × 10^−3^): training learning rate

Our selection of hyperparameters is bolded above.

We train for a maximum of 100 epochs, with early stopping (patience = 10) based on validation gene (*n* = 2,126) Pearson correlation. We use Adam optimizer with weight decay=1 × 10^−5^, learning rate scheduler ‘cycle’ with base_lr = learning_rate/2, max_lr=learning_rate × 2, cycle_momentum = false. We use gradient clipping with gradient_clip_val = 1, ReLU activation functions and batch size of 16.

### P-SAGE-net training

P-SAGE-net is trained on ROSMAP training individuals and genes from a specified gene set that are in training chromosomes. It takes as input reference and personal sequence (phased to produce two haplotypes) and predicts two outputs: mean expression (across ROSMAP training individuals) and individual gene expression *z*-score (with respect to training individuals). All model hyperparameters are the same as for r-SAGE-net. In addition to the convolutional and pooling layers of r-SAGE-net, p-SAGE-net also has one internal fully connected layer that the flattened personal and reference tensor pass through and separate output heads of fully connected layers to predict the two outputs (see Supplementary Fig. 8 for an architecture diagram). Each epoch entails training on all training genes and training individuals and validating on all validation genes and validation individuals. When p-SAGE-net is initialized from r-SAGE-net, all parameters in the convolutional and pooling layers of r-SAGE-net are loaded into p-SAGE-net. We train for a maximum of 25 epochs for the ‘best’ p-SAGE-net model shown in Fig. 1 and ten epochs for all other model variations, selecting best epoch based on train gene per-gene Pearson correlation. We use batch size of 6. The model training procedure is the same for the DNAm analysis, except the DNAm p-SAGE-net models are only trained for a single epoch, due to compute constraints and our intention to focus on unseen-region performance. As shown in Extended Data Fig. 8, while seen-region performance does increase with training epoch, unseen-region performance does not.

### P-SAGE-net ablation experiments

For ‘non-z-score’, instead of predicting individual *z*-score, we instead predict difference between mean expression (across training individuals) and the individual’s gene expression. For ‘transformer block’, the last convolutional block (at the lowest resolution) is replaced with a transformer block (with n_filters = kernel_number, nhead = kernel_size). For ‘non-contrastive’, instead of splitting personal gene expression prediction into mean and difference, the model is trained to predict a single output (an individual’s gene expression) from the individual’s personal genome sequence.

### Predicting gene expression with Enformer

We use GTEx mean gene expression to assign weights to each of Enformer’s 5,313 human output tracks. To go from Enformer’s outputs to fine-tuned predictions, we use these weights to transform the log(prediction + 1) of Enformer’s three center bins (447,448,449). We then sum these three center bins to get the final fine-tuned prediction. We use these fine-tuned predictions for all analyses using Enformer. For the non-fine-tuned version shown in Extending Data Fig. 1c, we take log(prediction + 1) of Enformer’s three center bins (447,448,449) for this track and sum. We load the Enformer model from https://tfhub.dev/deepmind/enformer/1 and use model.predict_on_batch to make predictions.

### Model attributions

To identify seqlets in our model attributions, we use the tangermeme recursive_seqlets simplification of TF-MoDISco^25^ with additional_flanks = 2. We use the tangermeme annotate_seqlets implementation of TOMTOM^25^ to match motifs to the HOCOMOCO v.12 database^26^. To perform ISM with p-SAGE-net, we mutate both haplotypes of the personal sequence and record the difference output of the model for each mutated base. We zero-center both ISM and gradients by subtracting the mean at each position^27^. For the DNAm global attribution analysis, we identify seqlets from r-SAGE-net and p-SAGE-net zero-centered gradients. For clustering, we consider all seqlets with *P* < 0.005. We obtain a metric of motif-motif similarity using the function torch_compute_similarity_motifs from https://github.com/sasselab/DRG/blob/main/drg_tools/motif_analysis.py, metric = correlation_pvalue, padding = 0, bk_freq = 0, reverse_complement = True. We then use this similarity metric as input to sklearn AgglomerativeClustering (linkage = complete, distance_threshold = 0.05) to determine motif clusters (we cluster p-SAGE-net and r-SAGE-net clusters together). We match these clusters to the HOCOMOCO v.12 database, accounting for multiple testing using the Benjamini–Hochberg procedure (reported as TOMTOM q-values).

To identify the enriched p-SAGE-net and r-SAGE-net clusters in Extended Data Fig. 9, we use one-sided Fisher’s exact test on 2 × 2 contingency tables of seqlet counts inside versus outside each cluster (Extended Data Fig. 9c,d). *P* values were corrected for multiple testing using the Benjamini–Hochberg procedure. We show clusters that are both significantly enriched for the given model and significantly match to the motif database (TOMTOM *q* < 0.05). To select the ten ‘top clusters’ for p-SAGE-net, r-SAGE-net and both models combined in Extended Data Fig. 10 we filter for clusters where both the number of seqlets and the mean absolute seqlet attribution are above their respective median values. We then sort by cluster-to-database TOMTOM *q*-value and show the ten most significant matches from this set.

### Reporting summary

Further information on research design is available in the Nature Portfolio Reporting Summary linked to this article.

## Online content

Any methods, additional references, Nature Portfolio reporting summaries, source data, extended data, supplementary information, acknowledgements, peer review information; details of author contributions and competing interests; and statements of data and code availability are available at 10.1038/s41592-026-03124-8.

## Supplementary information

Supplementary Information Supplementary Figs. 1–8.

Reporting Summary

## Data Availability

Genotype, RNA-seq and DNAm data for the ROSMAP samples are available from the Synapse AMP-AD Data Portal (accession code syn2580853) as well as the Rush Alzheimer's Disease Center Research Resource Sharing Hub at www.radc.rush.edu. GTEx genotype and RNA-seq data are available from dbGaP under accession number phs000424.vN.pN.

## Code availability

Code to reproduce our analyses and instructions for using the software package that we describe is available at https://github.com/mostafavilabuw/SAGEnet and archived on Zenodo at 10.5281/zenodo.19961797 (ref. ^28^).
