# Supplementary material for: A scalable approach to investigating sequence-to-function predictions from personal genomes
Source: Nat Methods. 2026 Jun 8;23(7):1308–12. doi: 10.1038/s41592-026-03124-8 (PMC13345963; doi:10.1038/s41592-026-03124-8)
Supplement: Supplementary file 1 — Supplementary Figs. 1–8. [file 41592_2026_3124_MOESM1_ESM.pdf]

nature methods

Supplementary information https://doi.org/10.1038/s41592-026-03124-8

A scalable approach to investigating sequence-to-function predictions from personal genomes

In the format provided by the authors and unedited

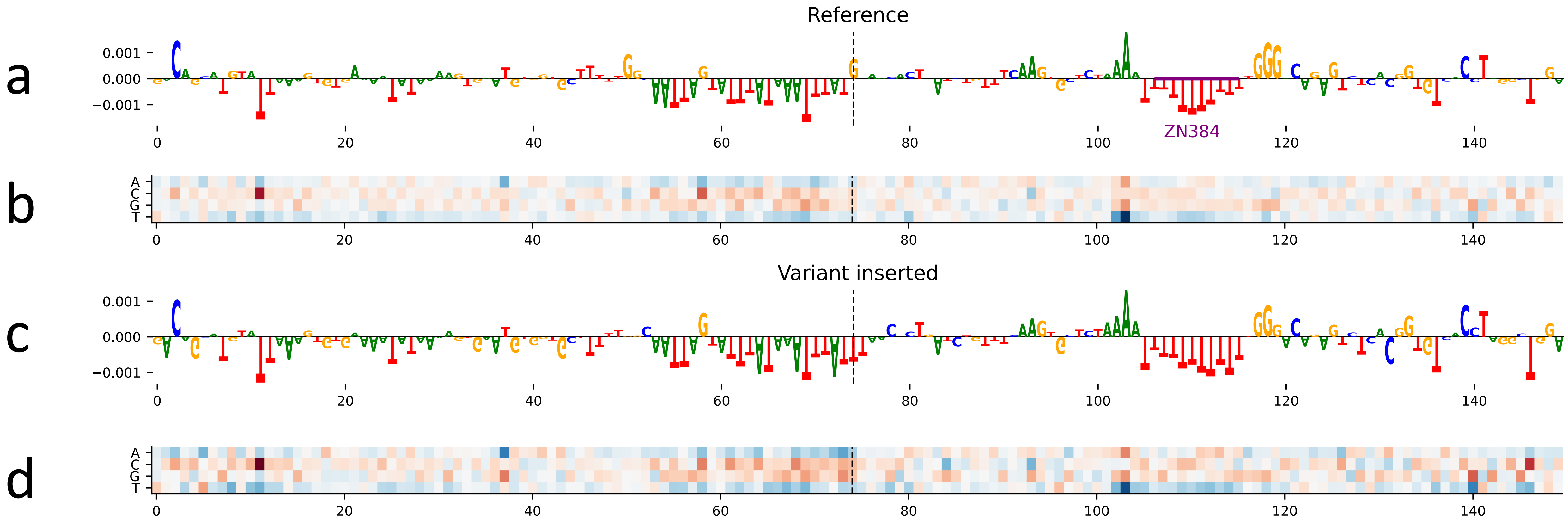

Supplementary Figure 1: Enformer ISM for GSTM3. Same analysis as Extended Data Fig. 3, but using fine-tuned Enformer instead of p-SAGE-net.

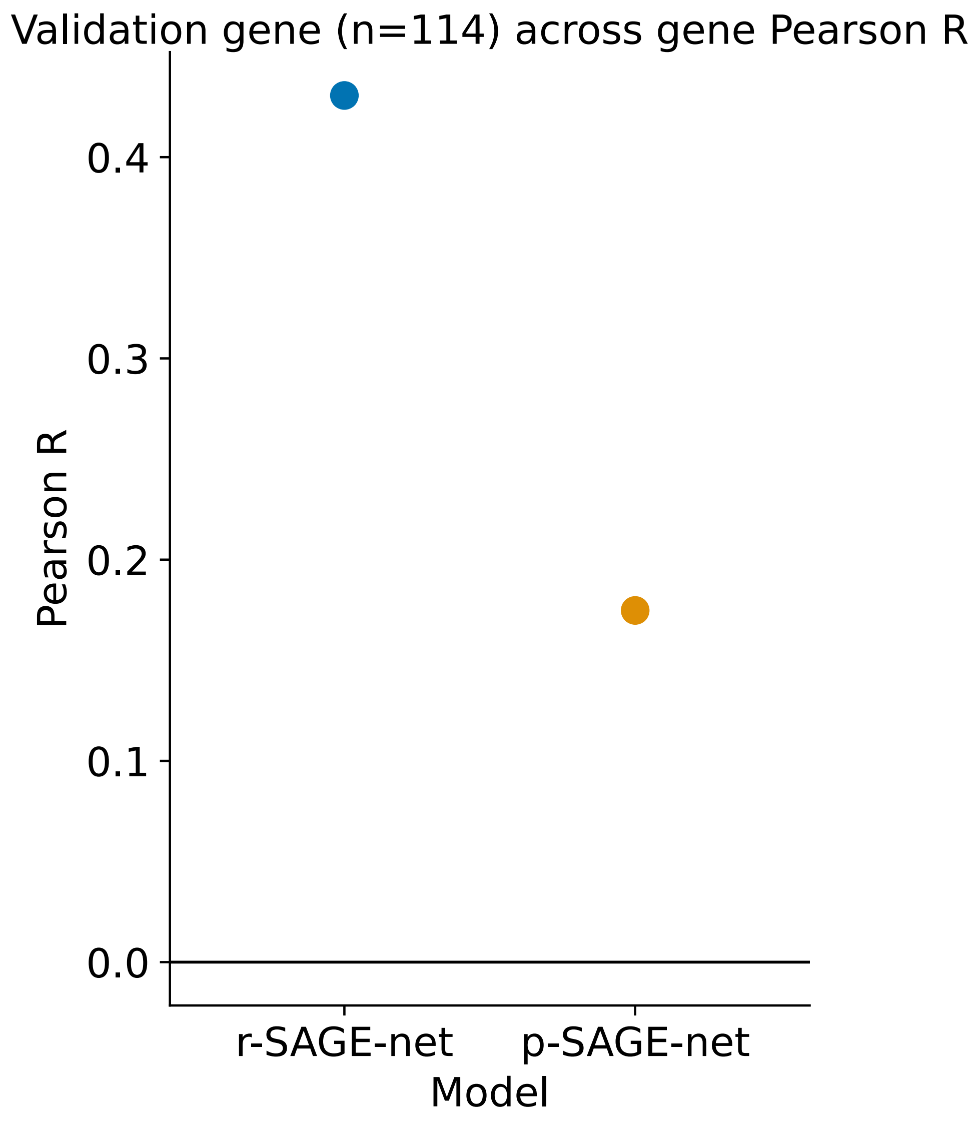

Supplementary Figure 2: Validation gene across-gene performance for p-SAGE-net and r-SAGE-net. 114 validation genes are from the top 1000 gene set used in Fig. 1c,d. P-SAGE-net and r-SAGE-net models are the same as in Fig. 1c,d. Pearson R is calculated with respect to ROSMAP population mean observed gene expression.

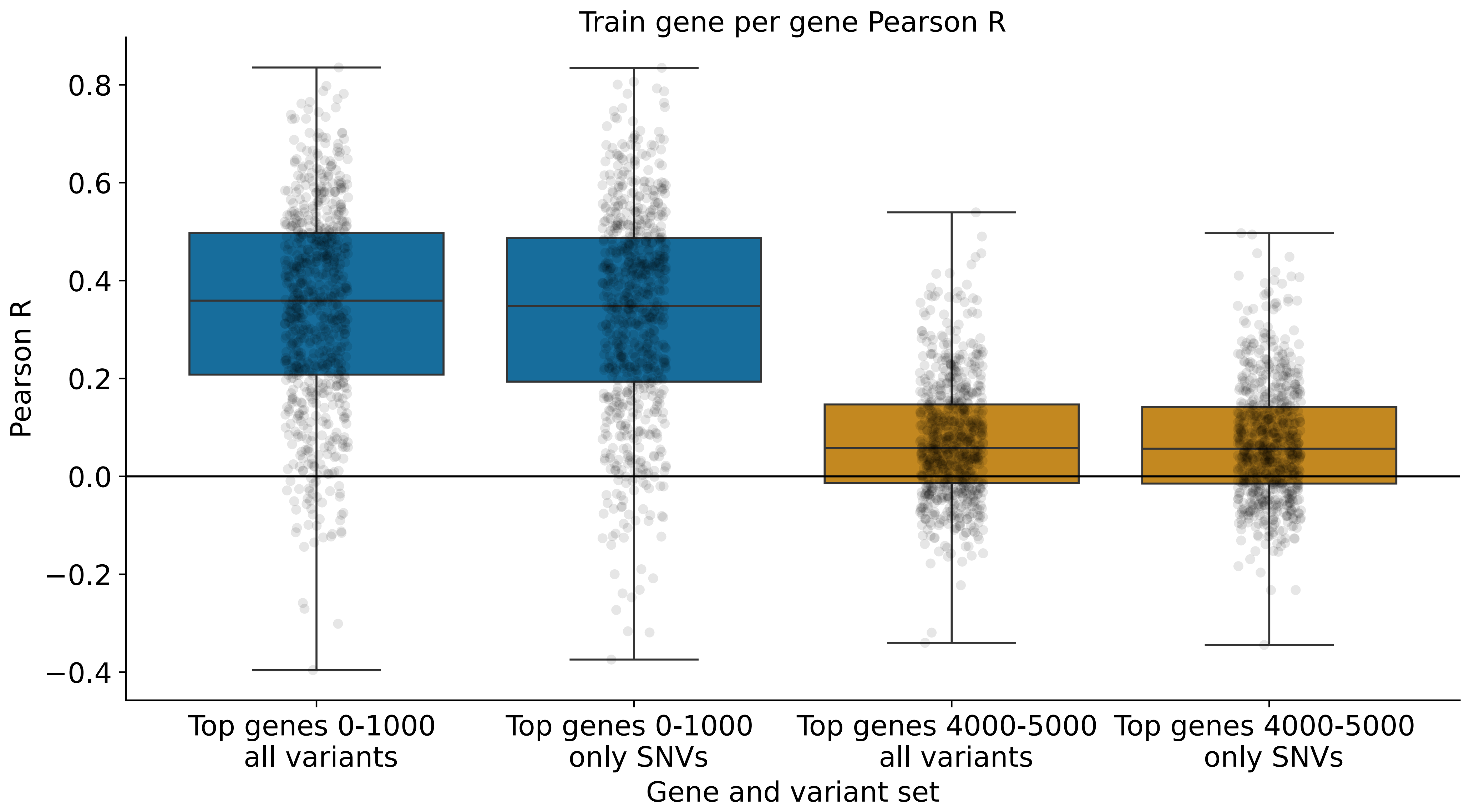

Supplementary Figure 3: Model evaluation using all variants using all variants vs. using only SNVs across two gene sets. Model and gene sets are the same as in Fig. 2b,c (0-1000, n=734, 4000-5000 n=756). For each gene set, the label "all variants" means that all variants present in the VCF are inserted, including indels. The label "only SNVs" means that only single nucleotide variants are inserted. These variant sets refer to model evaluation, not model training—the models were trained using all variants. Between top genes 0-1000 all variants and top genes 0-1000, two-sided Wilcoxon signed-rank test p=0.0228. Between top genes 4000-5000 all variants and top genes 4000-5000, two-sided Wilcoxon signed-rank test p=0.616. Each dot is one gene, boxplots are centered at median with top and bottom showing interquartile range and whiskers extending to minimum and maximum.

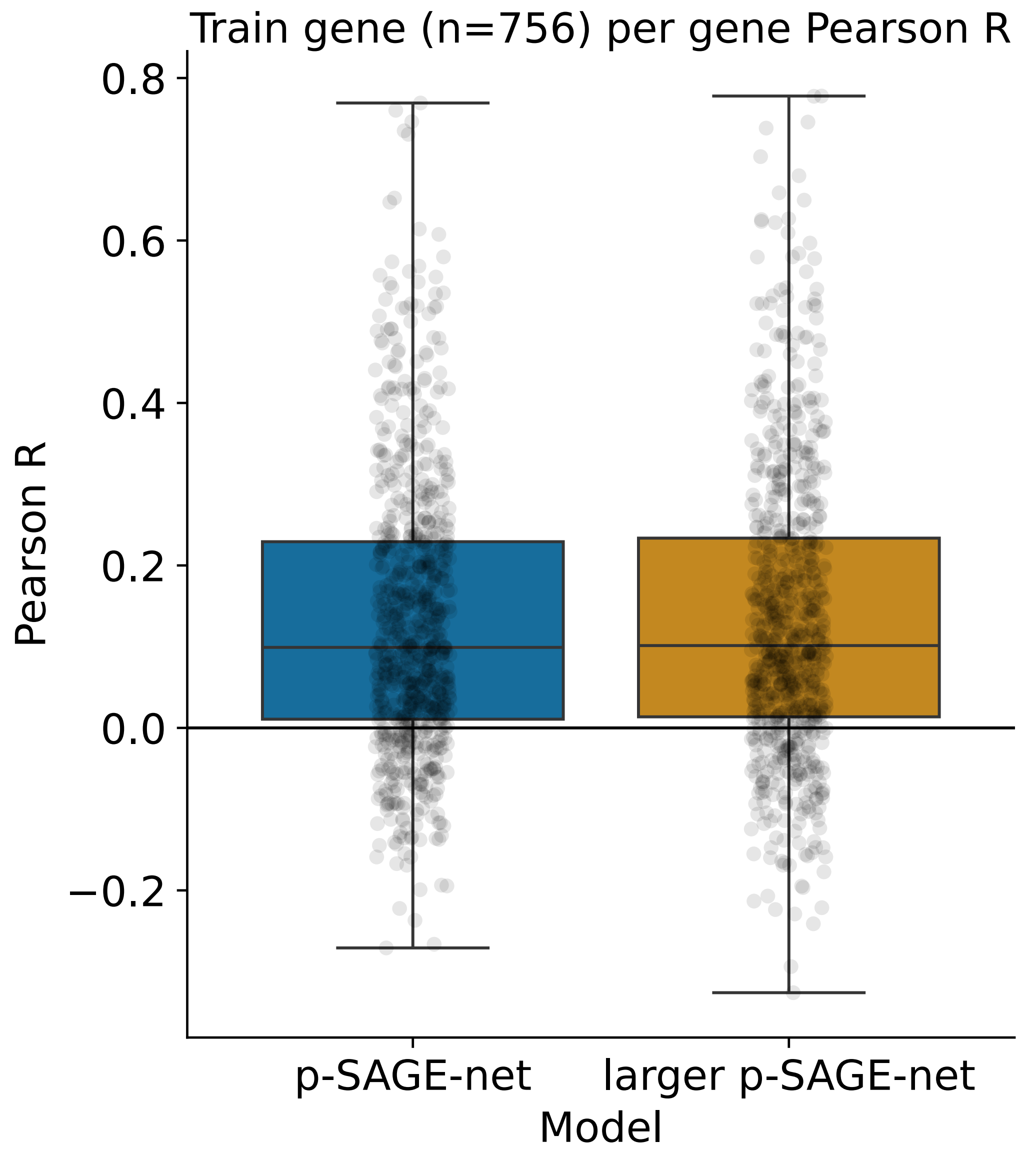

Supplementary Figure 4: Training on a larger gene set with increased model capacity. The evaluation shown for p-SAGE-net is the same as Fig. 2d, "Random genes", gene set size=3000. The evaluation shown for "larger p-SAGE-net" is the same but for a model with double the number of convolutional kernels in all convolutional layers after the first (512 instead of 256 kernels). Between p-SAGE-net and larger p-SAGE-net, two-sided Wilcoxon signed-rank test p=0.227. Each dot is one gene, boxplots are centered at median with top and bottom showing interquartile range and whiskers extending to minimum and maximum.

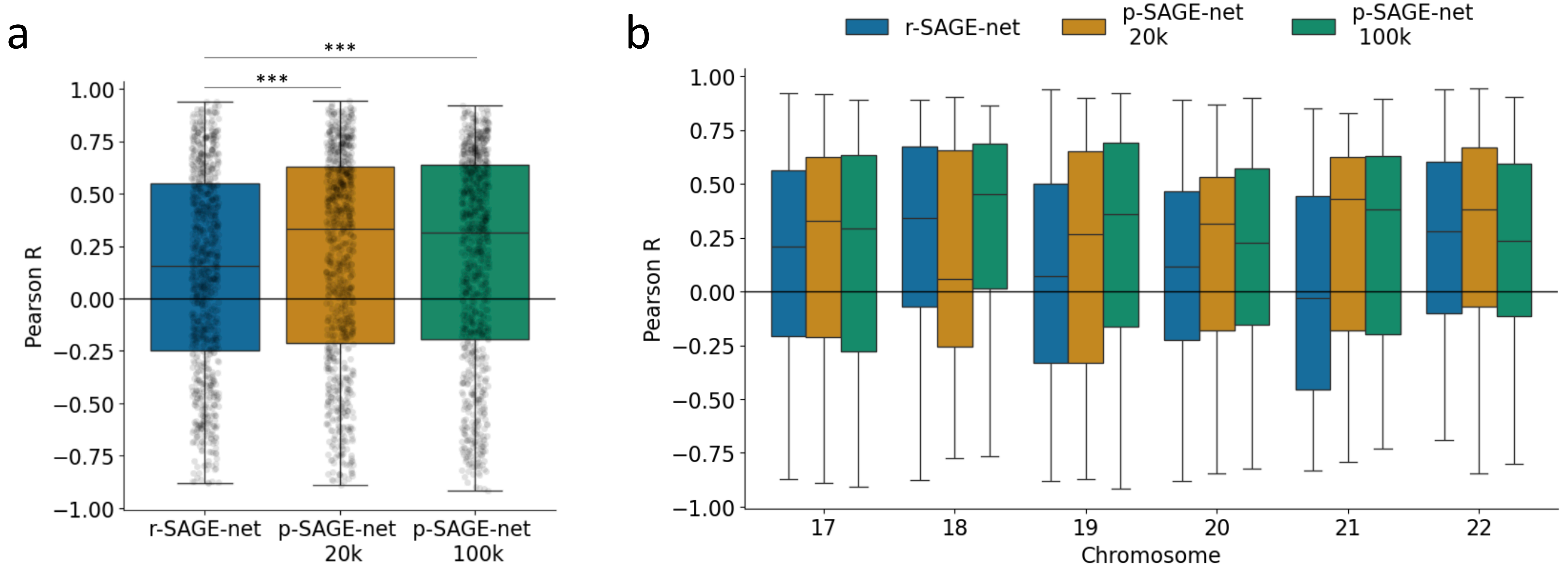

Supplementary Figure 5: DNAm r-SAGE-net, p-SAGE-net 20k, and p-SAGE-net 100k performance across all evaluation chromosomes. (a) Same evaluation as in Fig. 2f, but shown for all evaluation chromosomes (instead of only test chromosomes), n=2124. Significance shown is from one-sided Wilcoxon signed-rank test to r-SAGE-net performance (p=8.30e-5 for p-SAGE-net 20k, p=7.67e-04 for p-SAGE-net 100k. Each dot is one region, boxplots are centered at median with top and bottom showing interquartile range and whiskers extending to minimum and maximum. (b) Same evaluation as (a), but shown split across evaluation chromosomes. Boxplots are centered at median with top and bottom showing interquartile range and whiskers extending to minimum and maximum.

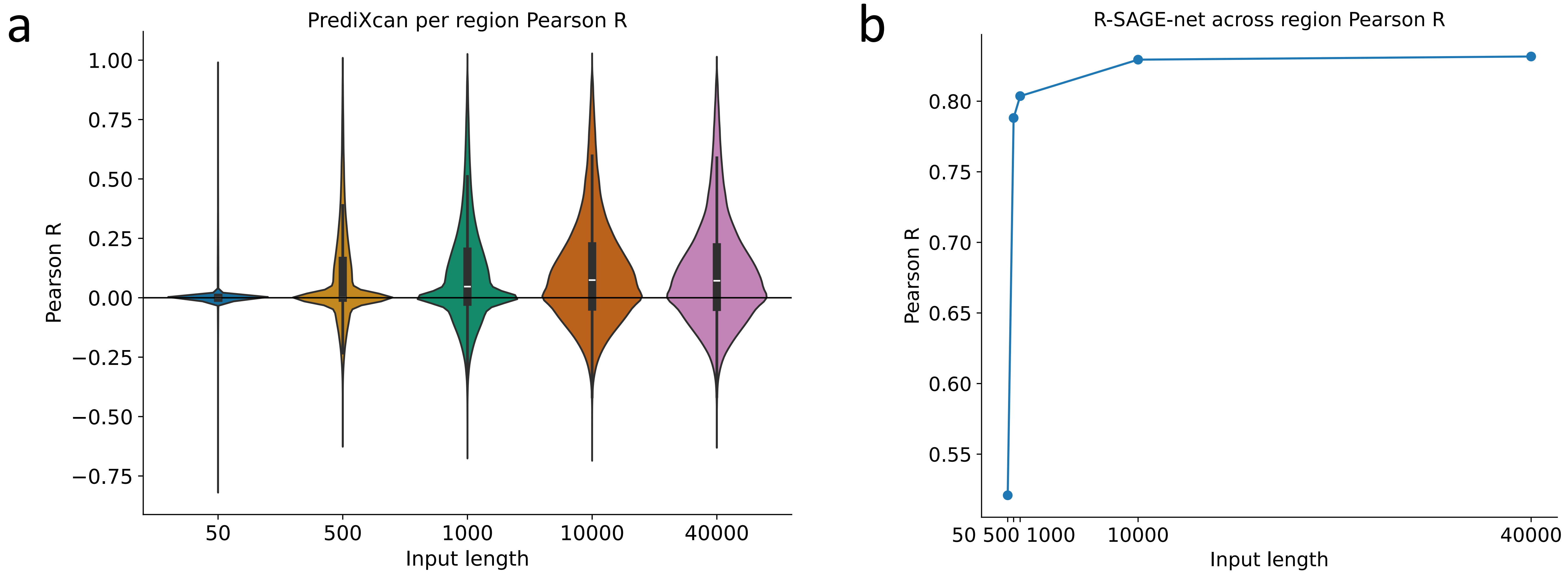

Supplementary Figure 6: DNAm PrediXcan and r-SAGE-net performance by input length. (a) PrediXcan per-region Pearson R across 54 test individuals for 95,000 randomly samples regions. Violin plots show the full distribution of the data; inner box is centered a median with top and bottom showing interquartile range (IQR) and whiskers extending to 1.5 x IQR. (b) R-SAGE-net across-region Pearson R across 5000 randomly sampled test regions. Pearson R is calculated with respect to population mean observed DNAm.

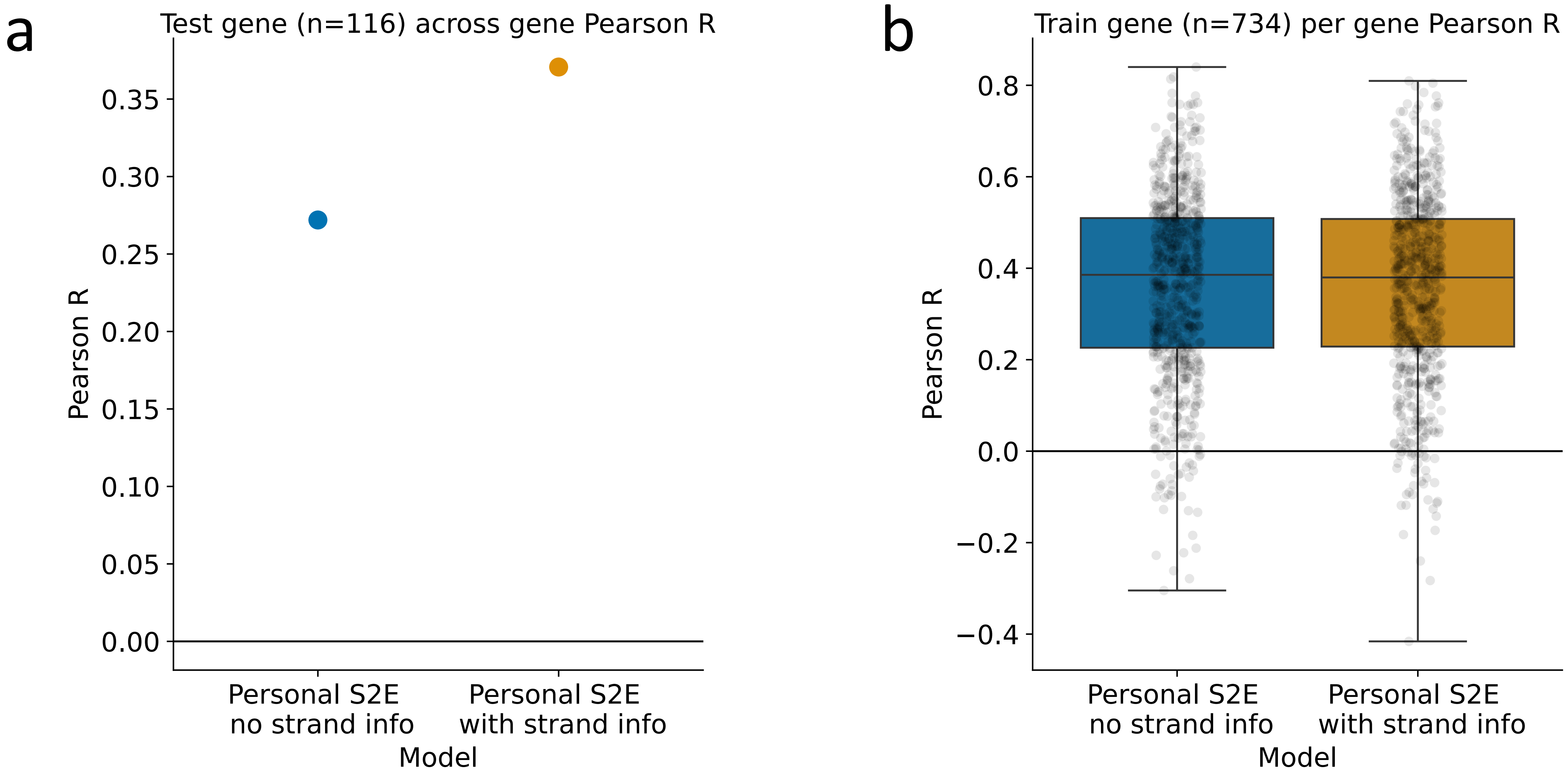

Supplementary Figure 7: Effect of using gene strand information. (a) Test gene across-gene Pearson R from 116 test genes from the top 1000 set. Pearson R is calculated with respect to ROSMAP population mean observed gene expression. (b) Train gene per-gene Pearson R across 205 GTEx individuals for 734 train genes from the top 1000 set. For "with strand info", we take the reverse complement of the sequence for genes on the negative strand, while for "no strand info" we do not. Between no strand info and with strand info, two-sided Wilcoxon signed-rank test pp p=0.965. Each dot is one gene, boxplots are centered at median with top and bottom showing interquartile range and whiskers extending to minimum and maximum.

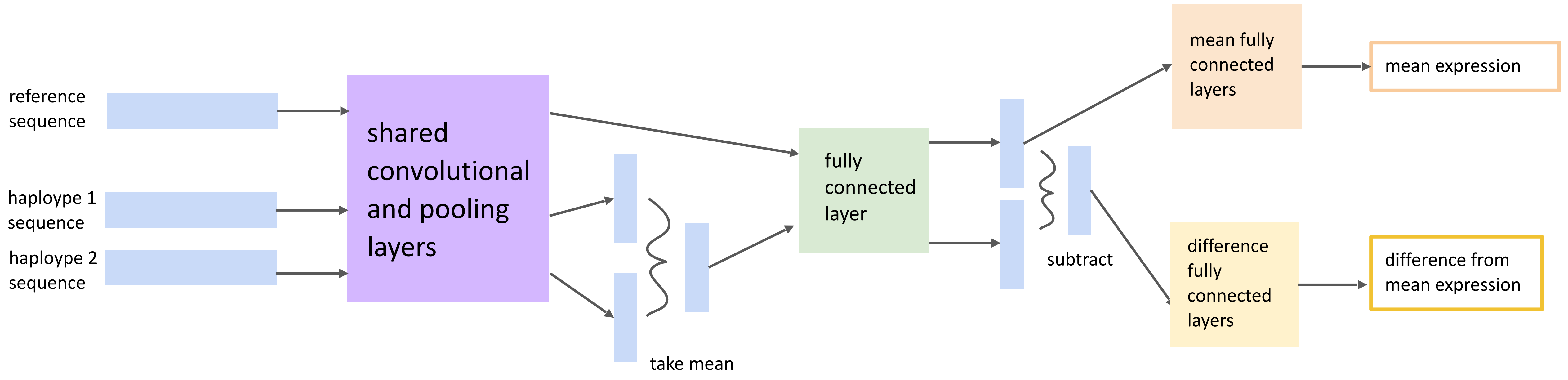

Supplementary Figure 8: P-SAGE-net model architecture. Model input is reference sequence for a given gene as well as an individual's two h aplotypes for that gene. These three sequences pass through the same shared convolutional layers, after which the two haplotypes are aver aged. The averaged personal tensor and the reference tensor then pass through the same fully connected layer and different output heads to produce mean expression and difference from mean expression. Mean expression is predicted from reference sequence alone, while difference from mean expression is predicted using all three sequences, specifically by subtracting the "reference" tensor intermediate output from the "personal". See Methods for model layer specifics.
