# Supplementary material for: A scalable approach to investigating sequence-to-function predictions from personal genomes
Source: Nat Methods. 2026 Jun 8;23(7):1308–12. doi: 10.1038/s41592-026-03124-8 (PMC13345963; doi:10.1038/s41592-026-03124-8)
Supplement: Supplementary file 2 — Reporting Summary [file 41592_2026_3124_MOESM2_ESM.pdf]

nature portfolio

Corresponding author(s): Sara Mostafavi

Last updated by author(s): 01/26/2026

Reporting Summary

Nature Portfolio wishes to improve the reproducibility of the work that we publish. This form provides structure for consistency and transparency in reporting. For further information on Nature Portfolio policies, see our Editorial Policies and the Editorial Policy Checklist.

Statistics

For all statistical analyses, confirm that the following items are present in the figure legend, table legend, main text, or Methods section.

n/a	Confirmed	
☐	☒	The exact sample size (*n*) for each experimental group/condition, given as a discrete number and unit of measurement
☒	☐	A statement on whether measurements were taken from distinct samples or whether the same sample was measured repeatedly
☐	☒	The statistical test(s) used AND whether they are one- or two-sided<br>*Only common tests should be described solely by name; describe more complex techniques in the Methods section.*
☒	☐	A description of all covariates tested
☐	☒	A description of any assumptions or corrections, such as tests of normality and adjustment for multiple comparisons
☐	☒	A full description of the statistical parameters including central tendency (e.g. means) or other basic estimates (e.g. regression coefficient) AND variation (e.g. standard deviation) or associated estimates of uncertainty (e.g. confidence intervals)
☒	☐	For null hypothesis testing, the test statistic (e.g. *F*, *t*, *r*) with confidence intervals, effect sizes, degrees of freedom and *P* value noted<br>*Give P values as exact values whenever suitable.*
☒	☐	For Bayesian analysis, information on the choice of priors and Markov chain Monte Carlo settings
☒	☐	For hierarchical and complex designs, identification of the appropriate level for tests and full reporting of outcomes
☐	☒	Estimates of effect sizes (e.g. Cohen's *d*, Pearson's *r*), indicating how they were calculated

Our web collection on statistics for biologists contains articles on many of the points above.

Software and code

Policy information about availability of computer code

Data collection	No software used in data collection.
Data analysis	The coordinates of variant calls (GRCh37) were converted to GRCh38 coordinates using the Picard LiftoverVcf tool (http://broadinstitute.github.io/picard). We use the Eagle software version 2.4.1 to phase the genotypes with the default setting. We use Enformer software from https://tfhub.dev/deepmind/enformer/1. We use pysam 0.23.0 (https://pysam.readthedocs.io/en/latest/api.html) to construct our personal genome datasets and tangermeme (https://github.com/jmschrei/tangermeme) for seqlet analysis. Our software and scripts to reproduce our analyses can be found at: https://github.com/mostafavilabuw/SAGEnet/tree/main.

For manuscripts utilizing custom algorithms or software that are central to the research but not yet described in published literature, software must be made available to editors and reviewers. We strongly encourage code deposition in a community repository (e.g. GitHub). See the Nature Portfolio guidelines for submitting code & software for further information.

Data

Policy information about availability of data

All manuscripts must include a data availability statement. This statement should provide the following information, where applicable:

- Accession codes, unique identifiers, or web links for publicly available datasets
- A description of any restrictions on data availability
- For clinical datasets or third party data, please ensure that the statement adheres to our policy

Genotype and RNA-seq data for the Religious Orders Study and Rush Memory and Aging Project (ROSMAP) samples are available from the Synapse AMP-AD Data Portal (accession code syn2580853) as well as the RADC Research Resource Sharing Hub at www.radc.rush.edu. GTEx genotype and RNA-seq data are available from dbGaP (accession number phs000424.vN.pN).

Research involving human participants, their data, or biological material

Policy information about studies with human participants or human data. See also policy information about sex, gender (identity/presentation), and sexual orientation and race, ethnicity and racism.

Reporting on sex and gender	We consider sex as a biological variable in our preprocessing of RNA-seq data. We do not consider gender.
Reporting on race, ethnicity, or other socially relevant groupings	We do not consider race or ethnicity in our analyses.
Population characteristics	Population characteristics include age and sex.
Recruitment	This study did not collect any new data. See cited ROSMAP and GTEx studies for further information.
Ethics oversight	See cited ROSMAP and GTEx studies for further information.

Note that full information on the approval of the study protocol must also be provided in the manuscript.

Field-specific reporting

Please select the one below that is the best fit for your research. If you are not sure, read the appropriate sections before making your selection.

☒ Life sciences ☐ Behavioural & social sciences ☐ Ecological, evolutionary & environmental sciences

For a reference copy of the document with all sections, see nature.com/documents/nr-reporting-summary-flat.pdf

Life sciences study design

All studies must disclose on these points even when the disclosure is negative.

Sample size	We use 859 individuals from the ROSMAP cohort and 205 individuals from the GTEx cohort for our gene expression analysis. These are all individuals for whom we have access to WGS and cortex RNA-seq data. For our ROSMAP DNAm analysis we use 634 individuals (these are all the individuals for whom we have access to WGS and DNAm).
Data exclusions	No samples were excluded from our analyses.
Replication	N/A.
Randomization	ROSMAP individuals were split randomly into train/validation/test groups. Genes were split by chromosome.
Blinding	N/A.

Reporting for specific materials, systems and methods

We require information from authors about some types of materials, experimental systems and methods used in many studies. Here, indicate whether each material, system or method listed is relevant to your study. If you are not sure if a list item applies to your research, read the appropriate section before selecting a response.

Materials & experimental systems

n/a	Involved in the study
☒	☐ Antibodies
☒	☐ Eukaryotic cell lines
☒	☐ Palaeontology and archaeology
☒	☐ Animals and other organisms
☒	☐ Clinical data
☒	☐ Dual use research of concern
☒	☐ Plants

Methods

n/a	Involved in the study
☒	☐ ChIP-seq
☒	☐ Flow cytometry
☒	☐ MRI-based neuroimaging

Plants

Seed stocks	*Report on the source of all seed stocks or other plant material used. If applicable, state the seed stock centre and catalogue number. If plant specimens were collected from the field, describe the collection location, date and sampling procedures.*
Novel plant genotypes	*Describe the methods by which all novel plant genotypes were produced. This includes those generated by transgenic approaches, gene editing, chemical/radiation-based mutagenesis and hybridization. For transgenic lines, describe the transformation method, the number of independent lines analyzed and the generation upon which experiments were performed. For gene-edited lines, describe the editor used, the endogenous sequence targeted for editing, the targeting guide RNA sequence (if applicable) and how the editor was applied.*
Authentication	*Describe any authentication procedures for each seed stock used or novel genotype generated. Describe any experiments used to assess the effect of a mutation and, where applicable, how potential secondary effects (e.g. second site T-DNA insertions, mosiacism, off-target gene editing) were examined.*
